# Topography Influence on Noble Metals’ Work Function Measured In Vacuo by Photoelectron Spectroscopy and Kelvin Probe Force Microscopy

**DOI:** 10.1021/acsami.6c00932

**Published:** 2026-06-09

**Authors:** Artem M. Dmitriev, Marcin Kisiel, Akash Gupta, Laurent Marot, Ernst Meyer

**Affiliations:** Department of Physics, 27209 University of Basel, Klingelbergstrasse 82, CH-4056 Basel, Switzerland

**Keywords:** work function, gold, silver, photoelectron spectroscopy, Kelvin probe force microscopy, surface roughness, topography

## Abstract

The work function (WF), the minimum energy required to extract an electron from a material’s surface into a vacuum, is affected by the parameters of the surface of the material, such as the surface topography. In the literature, it has been shown that changes in roughness affect the WF of metals; however, no clear relation between these two parameters was formulated. The paper presents the dependence of the WF on surface roughness for gold and silver thin films deposited on stainless steel substrates with roughness in the range of nanometers to micrometers. The WF measurements were performed using in-vacuo ultraviolet photoelectron spectroscopy (UPS) and in-vacuo Kelvin probe force microscopy (KPFM), ensuring that the surface remained free from air contamination and oxidation. The topography of the samples was characterized using both 3D laser confocal microscopy and atomic force microscopy. A study of the WF dependence on topography parameters, amplitude and slope, revealed that WF decreases with increasing roughness. Depending on the choice of roughness parameter, a logarithmic or linear relationship was observed. This behavior is attributed to the relationship between amplitude and slope parameters, which was validated experimentally. The decrease of the WF with increasing roughness, according to the literature, is linked to the electronic redistribution of charges and potential step at the surface of a material, which gives rise to the surface dipole, which depends sensitively on the surface’s atomic arrangement, namely, a roughness increase. The WF measurements using in-vacuo UPS and KPFM, performed on two samples with different morphologies, showed similar results. Additionally, after exposure to air, WF measurements via KPFM revealed a reduction of 0.45 eV compared to vacuum conditions. Overall, these results evidenced the critical aspect of in-vacuo measurement for the WF.

## Graphical abstract

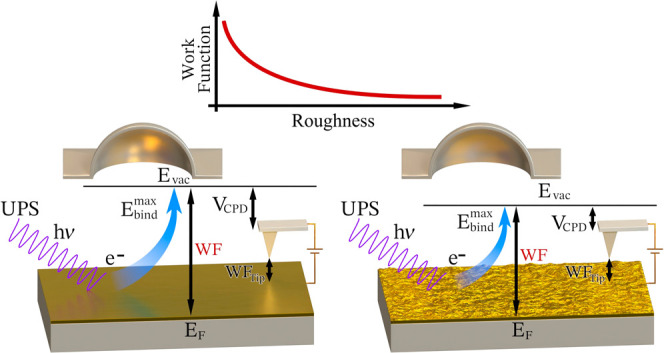

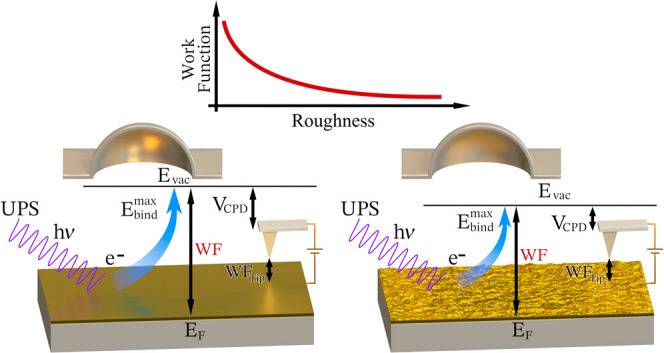

## Introduction

The work function (WF) is a fundamental property of materials that represents the minimum energy needed to remove an electron from a surface to a position in a vacuum. The WF is defined by the difference

$$\mathrm{WF}=E_{\mathrm{vac}}-E_{F}$$ 1

where *E*_vac_ is the electrostatic potential of the vacuum in the vicinity of the surface of the material, and *E*_F_ is the Fermi level of the material.

The WF is an intrinsic property of a material; however, it depends on a variety of its parameters. Generally, the WF is a combination of contributions from the bulk and the surface of the material. The bulk contribution is the energy difference between the electron bound and the vacuum level at an infinite distance.^1^ The electronic structure, corresponding to the material as well as to the defects and impurities, determines *E*_F_ and affects the resulting WF.^2^ The surface effect on the WF stems from the dipole distribution over the material’s surface.^3^ For instance, the same materials exhibit different WF depending on their surface state, structure, and crystal orientation.^4–6^

The WF is widely used by researchers to describe various processes occurring on surfaces, such as thermionic emission,^3^ corrosion,^7^ adsorption and desorption of atoms and molecules,^8,9^ adhesion,^10^ and gas detection.^11^ In electrochemistry, the WF of an electrode was found to be in a linear relation with the electrode potential during the process of the electrochemical reactions.^12^ Hence, by modification of the WF of the material, it is possible to control the surface behavior of the materials, which is crucial for the flow of the aforementioned processes.

The theory of WF calculation began with a Jellium model being a simple modelization of the periodic pseudopotential of metal surfaces.^13^ This advance of the WF theory was followed by the model of the stabilized jellium, which implemented a structureless pseudopotential and expanded the area of the model's applicability.^14^ A macroscopic theory of the WF in solids based on the dielectric formalism theory, which is applicable to a wide range of polycrystalline solids: metals, semiconductors, and dielectrics, is introduced by Fazylov.^15^ The most recent advances in the WF theory are linked to ab initio calculations.^16,17^ These studies are able to match the experimental results and predict the change of WF due to surface adsorption,^18^ alloying,^19^ and formation of nanostructures on the surface.^20,21^

In the literature, measurements of the WF have been performed based on several different approaches, such as thermionic emission, field emission, and photoelectron spectroscopy.^22^ The thermionic emission takes place when enough thermal energy is provided to a material, allowing electrons to overcome the WF of the material. The measurements of the thermoelectric current density and the temperature of the sample allow us to deduce the WF by solving the Richardson equation.^23^ A main disadvantage of implementing this method is the requirement of a material that can withstand heating at high temperatures.

Another method of WF measurement is Kelvin probe force microscopy (KPFM), which is based on the measurement of the relative contact potential difference (*V*_CPD_) of the surface by forming a capacitor between the sample and the reference electrode.^24^ By scanning the surface, the WF of the sample can be locally mapped and correlated with features of the sample’s surface. KFPM can also be conducted under different environmental conditions, as long as the probe WF remains unchanged. However, *V*_CPD_ does not provide any information about energy levels, including ionization energy and electron affinity.^25^

Additionally, WF can be determined via photoemission spectroscopy, which occurs when the energy of the incoming photon exceeds the WF of the material.^1^ In practice, a helium (He) ultraviolet light source is usually used to irradiate the surface of the sample, and the spectrum of the emitted photoelectrons is recorded using the electron analyzer. The minimum energy required to emit an electron from the surface into the vacuum equals the photon energy of the metal minus the cutoff energy of the photoelectron spectrum:^6^

$$\mathrm{WF}=h\nu -E_{\mathrm{cut}-\mathrm{off}}$$ 2

where *h*ν is the energy of the incoming photons emitted by the He discharge lamp.

It should be noted that the preparation conditions and the environmental history of the sample play a major role in the results of the measurements of the surface-related properties of materials such as the WF. For example, the surface of the materials can become oxidized or contaminated by adsorbed gases after contact with the atmosphere or long-term air storage. For instance, a polycrystalline gold thin film can exhibit a WF of 4.55 eV when exposed to ambient atmosphere and up to 5.35 eV when sputter-cleaned via argon ion bombardment and then analyzed under ultrahigh-vacuum (UHV) conditions.^25^

Surface roughness (SR) is a characteristic of surface topography that influences the mechanical, chemical, and electrical properties of materials.^2,26^ In the literature, it has been shown that changes in SR affect the WF of metals; however, no exact relation between WF and SR has yet been established. For example, air KPFM measurements have demonstrated that increasing SR leads to a decrease in the WF of metals such as copper and silver.^27,28^ Specifically, copper exhibited a power-law decrease with root-mean-square roughness,^28^ while silver showed a linear decrease with arithmetical mean roughness.^27^ Further studies using air KPFM measurements^29^ confirmed the power-law decrease of WF for copper and silver as a function of arithmetical mean roughness, while a linear increase in the WF of aluminum and magnesium alloys was reported. A decrease of the WF of gold nanoparticles was reported by Musa and Ghabboun^30^ for the arithmetical mean roughness in the range of 1.2–8.2 nm using KPFM measurements as well. A recent DFT study performed by Aghili et al.^31^ demonstrated the decrease of the gold WF with the magnitude of change depending on the arithmetical mean roughness.

In this study, we employed ultraviolet photoelectron spectroscopy (UPS) to investigate the link between SR and the WF of gold and silver thin films deposited by magnetron sputtering. The UPS measurements were performed without breaking the vacuum to avoid any uncertainties about the ambient conditions. A number of topographic parameters were used, such as arithmetical mean roughness, arithmetical mean slope roughness, root-mean-square roughness, root-mean-square slope roughness, spectrally relevant roughness, and the mean surface inclination angle. For two cases, the UPS results were cross-checked with in vacuo KPFM data. The results indicate that regardless of the SR definition, the WF decreases with increasing SR. However, depending on the chosen SR definition, the WF behavior with SR can be linear or can follow a logarithmic law.

## Materials and Methods

### Sample Preparation and Analysis

In order to obtain surfaces with different SR, the stainless steel substrates were initially abraded using *#*80 sandpaper and, in the step-by-step approach, polished by finer sandpapers (*#*220, 400, 600, 1200, and 2400) and polishing pastes (0.25 μm). The substrates were abraded in random directions over the surface in order to avoid any preferential directions during the WF measurements. One of the substrates was sandblasted to achieve a higher SR. A polished silicon (Si) wafer was used as a sample with the lowest SR (Si-1). After the surface processing, the substrates were cleaned in the ultrasonic bath with ethanol and acetone for 5 min. Since stainless steel samples were too large to fit in our KPFM facility, a silicon TipCheck sample (Budget Sensors Ltd.), which is used for AFM tip qualification and characterization, along with sample Si-1, was utilized as a sample for comparison between UPS and KPFM (Si-2).

Gold (Au) and silver (Ag) coatings were deposited by means of the magnetron sputtering technique on the substrates in a high vacuum facility pumped down to a base pressure of at least 2 × 10^–6^ Pa. Before each deposition, titanium was deposited in the vacuum chamber to capture the background gas and improve the vacuum conditions. The magnetron discharge was ignited at a pressure of 2 Pa for an argon (Ar) flow of 20 sccm and a distance target/substrate equal to 10 cm. The deposition rate was measured using Inficon XTM quartz crystal microbalance (QMB). The coating thickness was set to a target value of 15 nm based on the QMB measurements. It should be noted that, although the entire set of samples was coated with gold, only selected samples were deposited with silver to reduce the number of measurements. However, the number of silver-coated samples remained sufficient to provide a representative data set.

After each deposition, the absence of any contaminants on the surface of the samples was verified by X-ray photoelectron spectroscopy (XPS), and the WF was measured by UPS. To do so, the samples were transferred from the deposition chamber to the UHV XPS chamber without breaking the vacuum. The electron spectrometer was equipped with a hemispherical analyzer (Leybold EA10/100 MCD) and a monochromatized Al Kα X-ray source (*h*ν = 1486.6 eV). The normal electron escape angle and a step size of 0.05 eV were used. The binding energy scale was calibrated using a clean gold sample, and the position of the Au_4f7/2_ line was set at 84.0 eV binding energy. A helium discharge lamp emitting in the ultraviolet range (He I, *h*ν = 21.2 eV) with a spot size of a few mm^2^ was used for the UPS measurements. The Fermi level and the Au_4f7/2_ line of an Au sample were used for calibration.

The surface topography of the samples was characterized by using a three-dimensional laser scanning confocal microscope (3D LSCM, VK-X1100, Keyence). Additionally, the surface topography and contact potential difference (*V*_CPD_) between tip and sample were measured using an atomic force microscope (AFM, Nanosurf FlexAFM). The Nanosurf FlexAFM is designed for high-resolution surface characterization over relatively large lateral areas. The instrument uses a flexure-guided piezoelectric scanner that provides a maximum lateral scan range of 100 μm × 100 μm and a vertical range of approximately 10 μm, enabling both wide-area imaging and precise topographic measurements. The *Z*-axis is typically operated in closed-loop mode, ensuring high accuracy and minimal drift during spectroscopy and force measurements, while the lateral axes are commonly open-loop, allowing faster scanning. The optical beam deflection detection, based on a laser and quadrant photodiode, provides a wide detection bandwidth up to a few megahertz. The FlexAFM is controlled by a 24-bit high-resolution digital controller, enabling precise signal acquisition and real-time feedback control. During the LSCM measurements, objective lenses with 50 and 150 times magnification were used to reconstruct the surface topography over an area of 214 × 284 μm^2^ and 95 × 72 μm^2,^ respectively.

The in-vacuo AFM/KPFM, used for Si-1 and Si-2 WFa and topography measurement, operated in frequency modulation (FM) mode,^32^ where the constant amplitude *A* = 10 nm of the oscillating cantilever was controlled with a phase-locked loop electronic feedback system.^33^ During the topography measurements, KPFM images were acquired with the bias voltage applied to the sample, whereas the cantilever was kept grounded.^24^ KPFM measurements in UHV were performed in single-pass FM-KPFM mode, such that the topography and contact potential difference (CPD) were acquired simultaneously during scanning. The applied AC modulation voltage amplitude and frequency were equal to 100 mV and 330 Hz, respectively. A highly n-doped silicon cantilever characterized by the WF, WF_Si_ = 4.85 eV, with a super sharp tip of the height 10–15 μm and a typical tip radius equal to 2 nm (SSS-NCL from Nanosensors) was used. The oscillation frequency, spring constant, and quality factor under UHV conditions were equal: *f* = 180 kHz, *k* = 43 N m ^–1^, and *Q* = 20 × 10^3^, respectively. To avoid surface contamination, the transfer of the samples into the AFM microscope was performed under UHV conditions, using the vacuum suitcase with the base pressure in the range of 10^–8^ Pa.

### Topography Analysis

The most common way to define the SR is to calculate the arithmetical mean roughness (*R*_a_) by the following equation:

$$R_{a}=\frac{1}{N_{x}Ny}\sum_{i=1}^{N_{x}}\sum_{j=1}^{N_{y}}|z(i,j)|$$ 3

where *N*_*x*_ and *N*_*y*_ are the number of measures in the two directions and *z*(*i,j*) is the deviation of the surface profile from the mean line. Using the same data, it is possible to define as well the RMS roughness (*R*_q_):

$$R_{q}=\sqrt{\frac{1}{N_{x}Ny}\sum_{i=1}^{N_{x}}\sum_{j=1}^{N_{y}}z(i,j{)}^{2}}$$ 4

Another way to define roughness is to analyze the distribution of slopes over the entire surface profile. In this case, arithmetical mean slope roughness (*R*_Δa_) and RMS slope roughness (*R*_Δq_) can be calculated as follows:^26,34^

$$R_{\Delta a}=\frac{1}{\left(N_{x}-2)(N_{y}-2\right)}\sum_{i=1}^{N_{x}-2}\sum_{j=1}^{N_{y}-2}\Delta (i,j)$$ 5

$$R_{\Delta q}=\sqrt{\frac{1}{\left(N_{x}-2)(N_{y}-2\right)}\sum_{i=1}^{N_{x}-2}\sum_{j=1}^{N_{y}-2}\Delta (i,j{)}^{2}}$$ 6

where the slope Δ is given by

$$\Delta (i,j)=\sqrt{{\left(\frac{\partial z}{\partial x}(i,j)\right)}^{2}+{\left(\frac{\partial z}{\partial y}(i,j)\right)}^{2}}$$ 7

Based on these data, the mean inclination angle can also be calculated:

$$\delta_{m}=\frac{1}{N_{x}Ny}\sum_{i=1}^{N_{x}}\sum_{j=1}^{N_{y}}\mathrm{arc}\tan\sqrt{{\frac{\Delta z_{i}}{\Delta x}}^{2}+{\frac{\Delta z_{i}}{\Delta y}}^{2}}$$ 8

where Δ*z_i_*/Δ*x_i_* and Δ*z_i_*/Δ*y_i_* are, respectively, the slope due to a variation of height in the *x* direction and the *y* direction.

The surface topography can also be analyzed through the use of a power spectral density (PSD) function, which is the Fourier transform of the autocorrelation function that has been calculated from the surface profile.^36^ For example, the surface of the sample can be smooth at short wavelengths and rough at long wavelengths. To account for all the wavelengths of the PSD, the relevant roughness can be calculated as follows:

$$\sigma_{\mathrm{rel}}=\sqrt{2\pi \int_{f_{\min}}^{f_{\max}}\mathrm{PSD}(f)df}$$ 9

where *f*_min_ and *f*_max_ are correspondingly the minimum and the maximum frequencies of the analyzed PSD. The use of σ_rel_ allows describing the surface irregularities of different orders of magnitude.

For all studied samples, the SR was analyzed by calculation of the aforementioned quantities based on the 3-D surface profiles measured by LSCM and AFM.

## Results and Discussion

### Relationship of the Topography Parameters of LSCM and AFM

The topography images of the samples measured after Au deposition by means of LSCM are presented in Figure 1 for 50-× magnification. The samples Si-1 and Si-2 exhibited the smoothest surface (Figure 1a,b) and the lowest values of the topography parameters (Table 1). It should be noted, however, that the AFM measurements showed a difference in the topography of Si-1 and Si-2 (see A Comparison between UPS and KPFM section) since the Si-1 substrate was a silicon wafer and Si-2 had a nanostructured morphology, which is presented in Figure S1 of the Supporting Information (SI). The stainless steel substrates (Figure 1c–j) exhibited a topography characterized by random scratches. The depth of the scratches varied depending on the grade of sandpaper, with lower grades providing coarser topography. To achieve a higher roughness, one of the samples was sandblasted, which resulted in a different morphology pattern compared to the other samples (Figure 1j). After that, the topography of the samples was studied utilizing Nanosurf FlexAFM AFM, the results of which can be found in Figure S2 of the SI.

**Table 1:** Substrate Materials, Finishing Conditions, Surface Topography Parameters, and the WF of the Samples^a^

**substrate material**	Si		stainless steel							
**sample name**	Si-1	Si-2	1/4 μm	*#*2400	*#*1200	*#*600	*#*400	*#*220	*#*80	sandblasted
*R*_a_ (nm)	4	6	5	11	65	90	139	265	906	1340
*R*_q_ (nm)	6	8	7	15	88	119	195	393	1156	1648
*R*_Δa_ (rad)	0.004	0.028	0.009	0.04	0.266	0.205	0.394	0.298	0.566	0.485
*R*_Δq_ (rad)	0.016	0.035	0.036	0.071	0.385	0.272	0.572	0.393	0.777	0.668
δ_m_ (a.u.)	0.004	0.028	0.008	0.038	0.240	0.194	0.331	0.272	0.444	0.397
σ_rel_ (μm)	0.0007	0.010	0.011	0.032	0.154	0.098	0.248	0.143	0.385	0.352
Au WF (eV)	5.20	5.41	5.21	5.19	5.12	5.05	5.04	4.94	5.01	4.91
Ag WF (eV)	4.94					4.87		4.65	4.69	4.60

a The surface topography was measured after the deposition of the metallic films using 50× LSCM.

**Figure 1:**
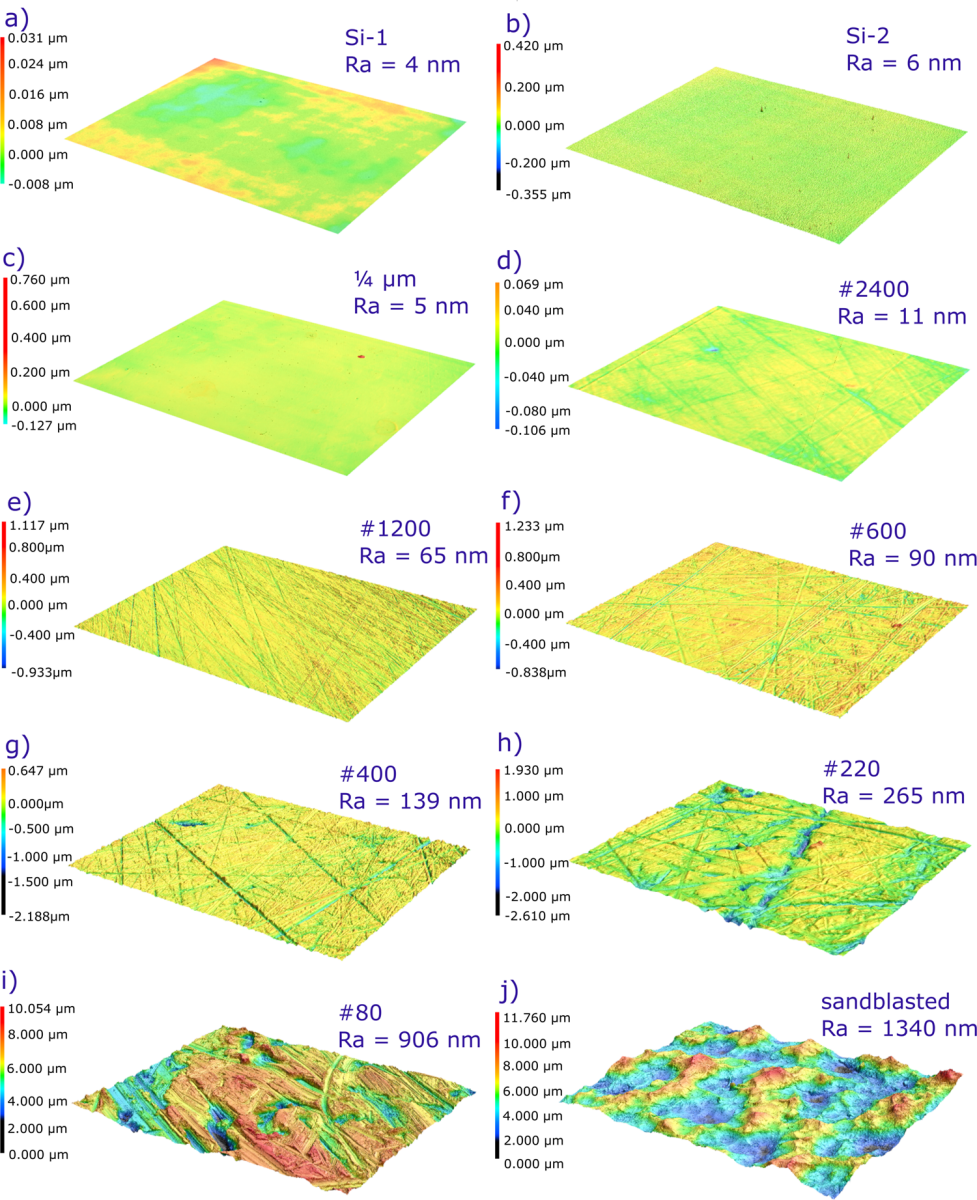
50× LSCM images of the samples: (a) Si-1 and (b) Si-2; stainless steel sample polished with (c) 1/4 μm diamond paste; stainless steel samples abraded with (d) *#*2400, (e) *#*1200, (f) *#*600, (g) *#*400, (h) *#*220, and (i) *#*80 sandpaper; and (j) sandblasted sample.

In order to determine which data will be used as the baseline for WF analysis, the topography parameters were calculated and compared utilizing topographical data obtained from both AFM and LSCM (50-× and 150-×) (Figure 2). Referring to Retailleau et al., we assumed standard deviations of 18 and 25% for the roughness parameters in AFM and LSCM, respectively.^35^

**Figure 2:**
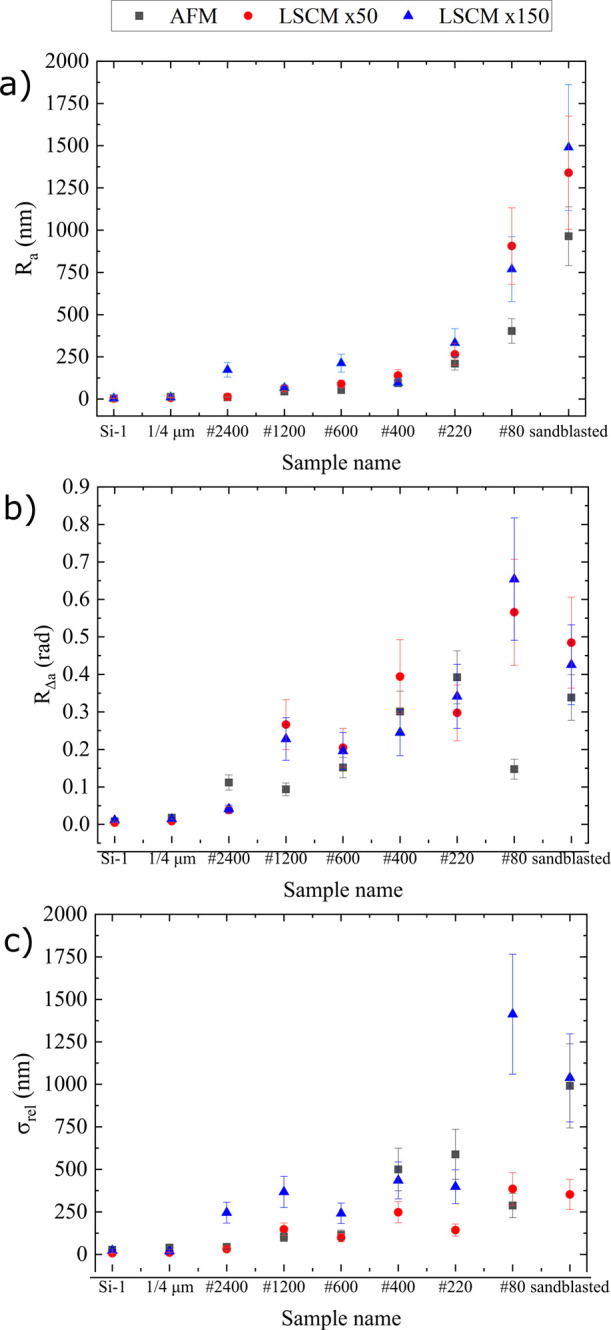
Comparison between the topography parameters: (a) arithmetical roughness, (b) arithmetical slope roughness, and (c) spectrally relevant roughness calculated using surface profiles measured using LSCM with magnifications ×50 and ×150 and AFM with an image size of 95 × 95 μm^2^. The error bars are assumed to be 18 and 25% for the roughness parameters in AFM and LSCM, respectively, referring to ref 35.

This difference between the data obtained using LSCM with x50 magnification and LSCM with ×150 magnification and AFM can be explained by the fact that for samples with higher roughness, the size of protrusions and valleys becomes comparable to the size of the image of both AFM and LSCM with ×150 magnification (95 × 95 μm^2^). For the last two samples, the AFM and ×150 LSCM images were smaller than the average size of features, leading to an underestimation of roughness values compared to those obtained with LSCM. Later on, the LSCM ×50 data, which was found relevant for both low and high roughness, was used as the main data set for further analysis of the relationship between the WF and SR. Table 1 gives the corresponding roughness parameters calculated using equations given in the Topography Analysis section and the value of Au and Ag WF for all the studied samples.

### Relationship Between WF and Topography Parameters

The WF was investigated for all of the samples after the deposition of gold and for selected samples after the deposition of silver and plotted as a function of different topography parameters, as shown in Figure 3. After each deposition and prior to the UPS analysis, XPS measurements were performed to ensure that no contaminants were present on the surface of the sample. The UPS and the XPS measurements of all of the studied samples are presented in Figure S3 of the SI.

**Figure 3:**
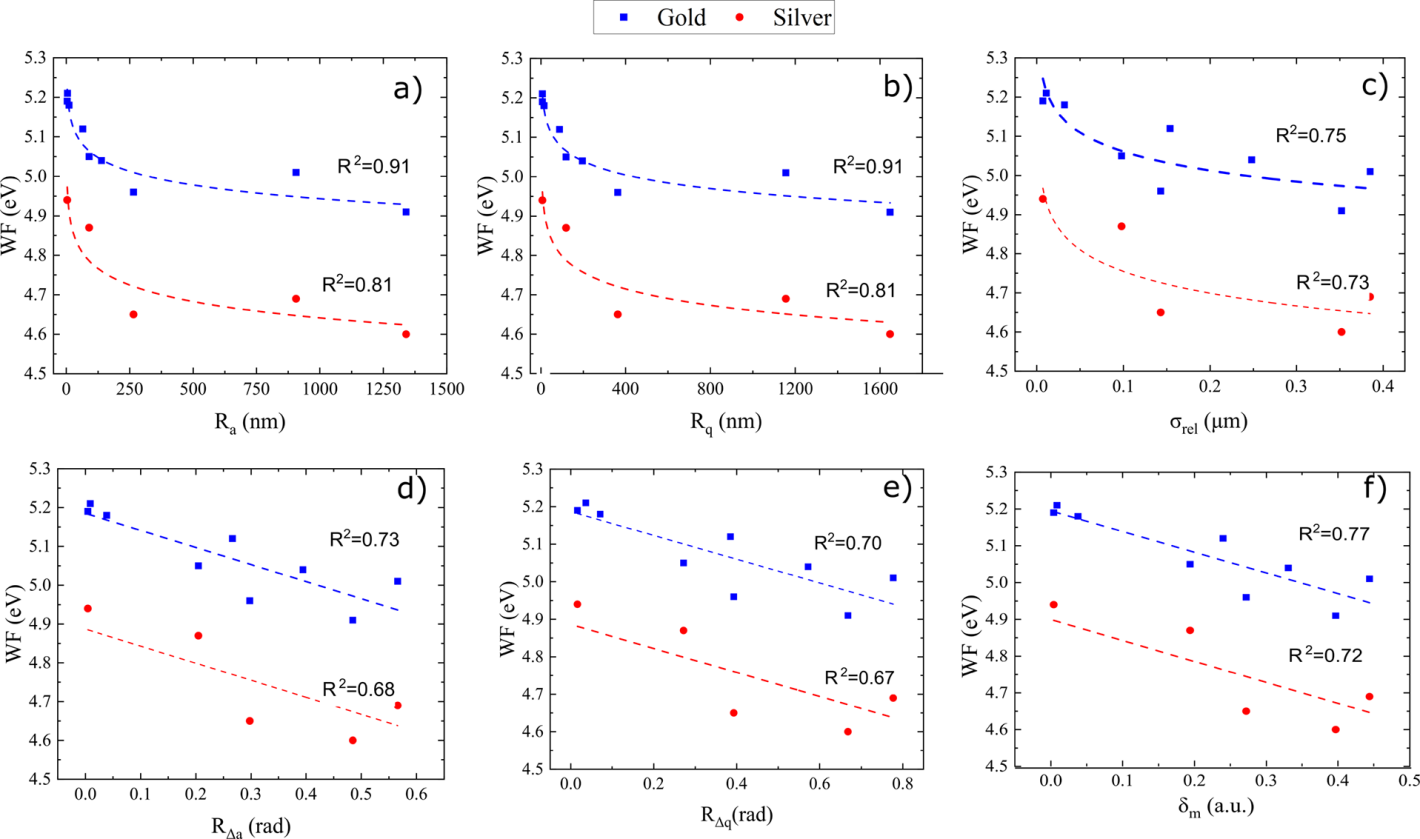
Measured WF of gold (blue squares) and silver (red circles) and corresponding fitting curves plotted as a function of *R*_a_ (a), *R*_q_ (b), σ_rel_ (c), *R*_Δa_ (d), *R*_Δq_ (e), and δ_m_ (f). Each plot includes a chi-squared (χ^2^) value, which quantifies the goodness of fit between the experimental data and the fitted model. Lower χ^2^ values indicate a better fit.

As illustrated in Figure 3, the WF decreases with increasing SR and the variation of WF with topography parameters exhibits two distinct behaviors. When the commonly used amplitude roughness parameters are used to describe the surface topography, the WF follows the logarithmic decrease (Figure 3a–c):

$$\mathrm{WF}=a-b\times \ln(R_{i})$$ 10

where *R*_i_ corresponds to a topography parameter (*R*_a_, *R*_q,_ or σ_rel_) and *a* and *b* are fitting parameters that are summarized in Table 2. Notably, the implementation of σ_rel_ as a topography parameter exhibits a similar manner of WF decrease, due to the fact that relevant roughness indicates the height change over all the frequencies of PSD.

**Table 2:** Parameters of the Logarithmic Fitting eq 10 for Gold and Silver WF

	gold		silver	
*R*_i_	*a*	*b*	*a*	*b*
*R*_a_	5.29	0.05	5.05	0.06
*R*_q_	5.30	0.05	5.07	0.06
σ_rel_	4.91	0.07	4.87	0.08

When the slope parameters are used to describe the surface topography, the WF exhibits a linear decrease with an increasing SR (Figure 3d–f):

$$\mathrm{WF}=c-d\times R_{i}$$ 11

where *R*_i_ is a slope roughness parameter (*R*_Δa_, *R*_Δq_, or δ_m_), and *c* and *d* are the fitting parameters that are summarized in Table 3.

**Table 3:** Parameters of the Linear Fitting eq 11 for Gold and Silver WF

	gold		silver	
*R*_i_	*c*	*d*	*c*	*d*
*R*_Δa_	5.18	0.44	4.88	0.45
*R*_Δq_	5.18	0.32	4.88	0.32
δ_m_	5.91	0.57	4.89	0.57

The goodness-of-fit of the model to the WF data is quantified using the chi-squared statistic (χ^2^), which was slightly lower for gold, primarily due to the larger number of data points and the greater scatter in the silver WF measurements. However, for both sets of data, χ^2^ was always found to be less than 0.1, which means that two simple fitting formulas (10) and (11) effectively characterize the measured data points. An additional set of statistical metrics presented in Tables S4 and S5 of Supporting Information exhibits values representing fine fitting of the data as well. From Tables 2 and 3, it is evident that the coefficients *b* in eq 10 and *d* in eq 11 are low, indicating a moderate decrease of WF variation with SR. The value of the coefficients *a* and *c* for eqs 10 and 11, respectively, corresponds to the maximum measured value of WF and is more likely to represent the WF of a smooth surface for both Au and Ag, which is close to the data of Si-1 in Table 1.

It should be noted that the slope topography parameters (*R*_Δa_, *R*_Δq_, or δ_m_) were recently demonstrated to be more representative of a surface compared to classically used amplitude parameters in the characterization of rough surfaces for predicting ion sputtering yield^37^ and Bidirectional Reflectance Distribution Function.^34^ In the case of the WF, based on the data presented in Figure 3, the use of these parameters instead of the classical amplitude ones does not provide any advantages in predicting the WF. Nevertheless, as noted earlier, WF correlations with *R*_Δa_, *R*_Δq_, and δ_m_ are linear, which can be convenient for WF prediction in practice.

The presence of two types of WF dependence on SR, logarithmic and linear, can be discussed using the example of the *R*_a_ and *R*_Δa_ pair. From eqs 10 and 11, one can deduce that the arithmetic slope roughness is related to the amplitude by an equation:

$$R_{\Delta a}=\frac{a-c}{d}+\frac{b}{d}\times \ln(R_{a})$$ 12

The plot of *R*_Δa_ as a function of *R*_a_, along with the data fitting using eq 12, is presented in Figure S6 of the SI. Similar patterns were observed for *R*_Δq_ and δ_m_ as a function of *R*_a_, as can be found in Figure S5. However, it is not clear if this dependence of amplitude and slope roughness is a general behavior or linked to a parameter surface profile *z*(*x, y*).

In summary, we observed a logarithmic dependence of WF on amplitude roughness parameters, while for angle and slope parameters, we have a linear dependence. This is in line with the studies of WFs on stepped surfaces, where it is found that the WF reduction is proportional to the step density. One can attribute a dipole moment to the edge atoms (typically in the range of 0.3 to 0.4 D per edge atom). As shown by Krahl-Urban et al., the step density follows a linear dependence on the inclination angle.^38^ Besocke et al. reported a linear decrease of the WF as a function of step density for Pt, Au, and W and interpreted it by attributing additional dipole moments to the atomic steps of the vicinal step structures.^39^ Therefore, we can conclude that the observed linear dependence on the mean inclination angle is a consequence of the dependence of the step density, which is then related to the dipole moments of the edge atoms, which reduce the WF. It should, however, be noted that Körner preferred to suppose a quadratic dependence on the inclination angle in contrast to our finding of a linear dependence.^40^

The nonlinear (logarithmic) dependence on the roughness *R*_a_ (amplitude) is also in line with theoretical calculations of Aghili et al.,^31^ where it is observed that the initial increase of roughness has a larger effect than the increase of roughness of subsequent layers. The fundamental parameter is the dipole moment of the edge atoms, which reduces the WFs of rough surfaces compared to the flat surfaces. Palasantzas showed that, for self-affine rough surfaces, the ratio of rough-surface to flat-surface potential depends on roughness parameters, paralleling the WF dependence on roughness observed here.^41^ Although their study focused on dielectric surfaces, it illustrates that evaluating roughness effects on surface potential is complex and simple models cannot capture the logarithmic WF behavior.

Additionally, a similar decrease in the WF with SR was previously reported in the literature. Xue et al.^29^ observed a similar trend for the WF of silver and copper as a function of *R*_a_, while Li and Li^28^ noted analogous behavior for copper WF relative to *R*_q_. Both studies employed air KPFM as the measurement tool and attributed the change in the WF with SR to variations in the capacitance between the Kelvin probe tip and the sample. Similarly, Wan et al.^27^ explained changes in the silver WF with SR through tip–sample capacitance but reported a linear relationship between WF and *R*_a_.

Moreover, the increase in the density of the states generated by the SR increase^42^ can trap the surface electrons, leading to a decrease in the overall electron density at the surface.^3^ A lower electron density can lead to a reduction in the WF as it is influenced by the minimal energy needed to remove an electron from the surface.^30^ Moreover, for Aghili et al.,^31^ higher SR is linked with a broader distribution of density of states and to a shift of the surface energy level to the *E*_F_. A combination of these factors leads to a decrease in the WF for the rough surfaces, which is observed in the present work, as well.

### Comparison Between UPS and KPFM

The WF comparison between two complementary techniques, UPS and KPFM, was performed using two gold-coated silicon substrates, one of which was a Si wafer (Si-1, Figure 1a) and the other one was a nanostructured TipCheck sample (Si-2, Figure 1b). The KPFM data for these two samples are shown in Figure 4. In Figure 4a,b, the 5 × 5 μm^2^ topography images are shown for the Si-1 and Si-2 surfaces, respectively. The corresponding KPFM images are shown in Figure 4c,d. A rather homogeneous KPFM contrast was observed across the measured surface, and *V*_CPD_ values were extracted along the diagonal profile lines. The extracted profile lines 1 and 2 for the Si-1 and Si-2 surfaces are shown in Figure 4e. The extracted contact potential difference *V*_CPD_ is equal to 0.570 ± 0.013 and 0.670 ± 0.010 V, leading to a difference of 0.1 V between the two films. It is worth mentioning that no tip change events were observed during the KPFM measurement. Moreover, *V*_CPD_ was independently determined for both surfaces by AFM force-spectroscopy, and the results are shown in Figure 4f. During the experiment, the cantilever was positioned at a few tip–sample distances (50, 40, 30, and 20 nm) and the sample bias voltage was swept from −0.5 to 1.5 V. The recorded shift of the cantilever resonance frequency versus bias voltage shows parabolic behavior, due to capacitive coupling of the tip and the underlying sample. The maximum of the parabola determines the *V*_CPD_ value. Again, the *V*_CPD_ shift equal to 0.1 V between Si-1 and Si-2 was measured, which is in agreement with the data shown in Figure 4e. No significant shift of *V*_CPD_ for different tip–sample distances was recorded, which again confirms homogeneous contact potential distribution across the surface. Moreover, KPFM measurements were performed at several sample locations and different scan sizes spanning from a few hundred nanometers squared to 20 μm^2^, again showing uniform CPD value across the surface.

**Figure 4:**
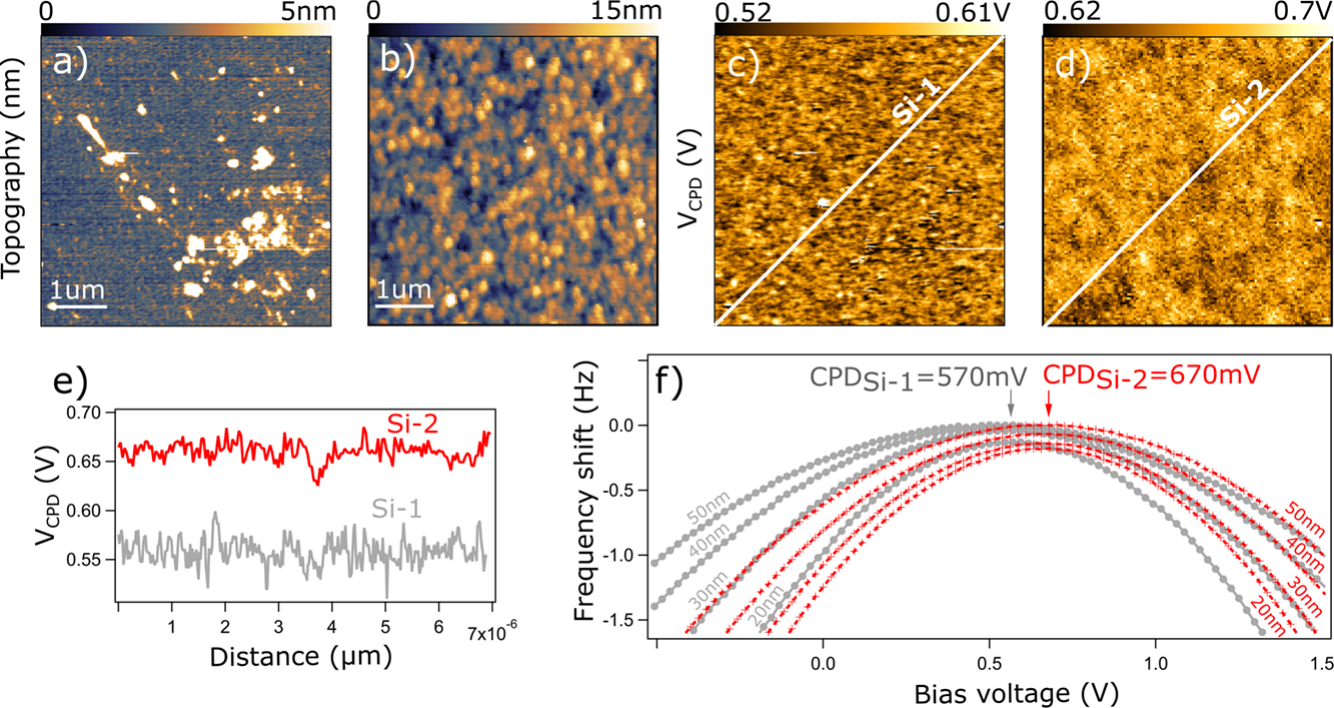
Noncontact AFM topography (a, b) and FM-KPFM (c, d) images taken under UHV conditions for the samples Si-1 (a, c) and Si-2 (b, d) coated with 15 nm of Au. The size of the scan frame is 5 × 5 μm^2^. The oscillation amplitude is 10 nm. In (e), the contact potential (*V*_CPD_) line profile data are shown, taken along the profile lines on C and D for the Si-1 (profile 1) and Si-2 (profile 2) surfaces, respectively. Additionally, the frequency shift parabolas versus sample bias voltage are shown in (f) for different tip–sample distances equal to *d* = 50, 40, 30, and 20 nm. The top of the parabola defines the value of contact potential equal to *V*_CPD_ = 570 and 670 mV for Si-1 and Si-2, respectively. The data are in agreement with the line profiles shown in (e).

Assuming the typical value of the WF of the silicon tip is equal to WF_Si_ = 4.85 eV,^43^ we can determine the WF of the measured sample:

$$\mathrm{WF}={\mathrm{WF}}_{\mathrm{Si}}+eV_{\mathrm{CPD}}$$ 13

and the results are summarized in Table 4, showing that both UPS and KPFM data taken under UHV conditions are in good agreement. Capacitance-related contributions to the KPFM signal can not be fully excluded. However, because the Si surface was coated with a 15 nm Au layer, voltage-dependent semiconductor capacitance effects are expected to be strongly suppressed, so the observed trend is more likely dominated by morphology-induced changes in the local electrostatic potential.

**Table 4:** Work Function Determined From UPS and KPFM Experiments under UHV and Ambient Conditions

	in vacuo		in air	*R*_a_ (nm)	
WF (eV)	UPS	KPFM	KPFM	LSCM	AFM
Si-1	5.24	5.42	5.02	4 ± 1	0.4 ± 0.05
Si-2	5.41	5.52	5.07	6 ± 1.5	8.85 ± 0.75

Surprisingly, the UPS and KPFM measurements show an increase (0.1–0.15 eV) of the WF for the structured Si-2 sample as compared to the sample Si-1. Since the measurements were performed under UHV conditions, no contaminants were observed on the surface of the samples, as confirmed by XPS. Thus, the WF variations are solely induced by the surface topography. One can expect that sharp conical structures of the sample Si-2 are supposed to alter the local electric fields, which slightly lowers the local barrier for electrons to escape into the vacuum and, hence, reduces the WF.^44,45^ It should be noted, however, that the cones of Si-2 were not sufficient to drastically increase the *R*_a_, which, according to AFM measurements in tapping mode, was equal to 8.8 nm, while the *R*_a_ of Si-1 was equal to 0.4 nm (see Figure S6). This suggests that the electric field enhancement is unlikely to be the primary cause of the WF change.

Although the roughness trends obtained from AFM and confocal microscopy are consistent, the absolute values vary with the measurement technique and scan size. This is expected because the two methods probe the surface morphology with different spatial resolution and sensitivity to high-aspect-ratio features. The measurement area for LSCM was 214 × 284 μm for magnification of 50X in comparison to AFM 5 × 5 μm. It has to be noted that, due to the larger image area in LSCM, clusters on the surface from the magnetron target are overestimated, leading to an overestimation of the Ra value. In particular, AFM may underestimate roughness on spike-like surfaces due to tip convolution effects and limited access to narrow depressions. Moreover, under ambient conditions (data shown in Figure S6 of the Supporting Information), water meniscus formation may further affect the apparent topography. The reported error bars, therefore, reflect both statistical variation and technique-dependent systematic uncertainties.

We propose to explain this increase of the WF for Si-2 as follows: (i) nanostructured Si with facet-oriented surfaces in the plane direction (111), (ii) a Au film with a preferential orientation of (111) related to compressive stress in the film, (iii) highest WF for (111) grain orientation, and (iv) increase of WF for the film having a compressive strain.

Nanostructured surfaces often consist of strain-stabilized microfacets with different crystallographic orientations. If these facets favor orientations with intrinsically higher WFs, the averaged WF can exceed that of a nominally flat surface, sometimes differing by tenths of an electronvolt.^46,47^ Recent studies have observed an increasing WF trend from (110) to (100) to (111) orientations,^48^ which was confirmed by Wang and Wang^49^ and Jooya et al.^50^ The latter study reported the following WFs for gold: Au(111), 5.60 eV, Au(100), 5.55 eV, and Au(110), 5.43 eV. Polycrystalline materials usually have lower WFs than single crystals.^51^

Additionally, in the literature, it was demonstrated that the stress induced in the coating during the deposition process also affects the WF. It was reported that the compressive in-plane strain on the Au(111) causes charge transfer in the system, causing the WF increase.^52^ A similar effect has been reported through DFT calculations by Umeno et al.^42^ for Au(111), (110), and (100) surfaces and by Chen et al. in ref 48 for Cu (100).

Similar gold films to Si-2 were deposited in our previous paper using the same parameters and exhibit a compressive stress of 30 MPa, and a (111) texture, which means that the majority of grains have their (111) planes aligned parallel to the sample surface.^53^ Additionally, pyramid-like structures on the Si-2 surface typically feature (111)-oriented facets,^54,55^ which may additionally enhance a (111) texture in thin films deposited on top of them. Therefore, the increase in WF observed for the structured Si-2 sample, compared to Si-1, is most likely explained by the interplay of the preferential (111) orientation of the Au coating combined with compressive strain, supported by previous work and literature. However, since texture and strain were not measured directly for this sample, this explanation remains tentative. Capacitance artifacts in KPFM are unlikely to dominate because the CPD was distance-independent (see Figure 4f). The parabolic frequency shift curves recorded at tip–sample distances of 20, 30, 40, and 50 nm reported CPD values that varied within the experimental uncertainty. The variation is below approximately 10–20 mV, whereas the CPD difference between Si-1 and Si-2 is 100 mV. This indicates that distance-dependent capacitive averaging is not sufficient to explain the observed WF difference. Moreover, the UPS data are not affected by the KPFM tip–sample capacitance. Since UPS and KPFM show the same trend, the effect is unlikely to be a KPFM capacitance artifact.

Finally, the samples Si-1 and Si-2 were exposed to air for 1 day, and their WF was measured again by means of KPFM. The AFM topography, KFPM images, and *V*_CPD_ taken by the air KPFM are presented in Figure S5. The WF of the samples under air condition was determined using AFM force-spectroscopy, which showed *V*_CPD_ = 0.17 V and *V*_CPD_ = 0.22 V for the Si-1 and Si-2 samples, respectively. The corresponding WF under ambient conditions (*T* = 23 °C and humidity of 60%) is equal to 5.02 and 5.07 eV, respectively. The reduction of the WF after the surface exposure to air, as compared to clean UHV conditions, is linked to the contamination of the surface of samples by the adsorbed gas molecules. In ref 25, a reduction of the gold WF by 0.6 eV is referenced, which is similar to a value of 0.45 eV reported in our contribution.

This result may explain the difference between different measurement results for the WF and the observation of the various trends of the WF and SR relationship, which are reported in the literature. Most of the corresponding studies have been conducted using air KPFM,^27–29,56^ where exposure to air can significantly influence WF measurements. In the case of noble metals such as gold, air exposure typically leads to a decrease in the WF due to gas adsorption on the surface. However, for metals that naturally form oxides, exposure to air can result in surface oxidation, leading to an increase in WF with increasing SR, as demonstrated in ref 56.

## Conclusions

The paper presents the results of the WF measurements performed using in-vacuo UPS of gold and silver samples with a roughness varying from 4 to 1340 nm. Stainless steel substrates were abraded with different sandpapers, and then gold and silver films were deposited on the surface using magnetron sputtering. Surface topography was analyzed using both 3D confocal microscopy and AFM measurements. Confocal microscopy, performed at ×50 magnification, was chosen as the primary method for topography analysis since, as compared to AFM, it provided similar results for low-roughness samples and more reliable measurements for high-roughness samples.

Based on the samples’ topography, surface roughness parameters such as *R*_a_, *R*_q_, *R*_Δa_, *R*_Δq_, and δ_m_ and σ_rel_ were calculated and the WF was plotted as a function of these parameters. For both Au and Ag, it was demonstrated that the WF always decreases with increasing SR. Depending on which type of topography parameters is used, amplitude (*R*_a_, *R*_q_, and σ_rel_) or slope (*R*_Δa_, *R*_Δq_, and δ_m_), the corresponding logarithmic or linear relationship of WF and SR was observed. Moreover, the relationship between amplitude and slope topography parameters was found to be logarithmic as well. The decrease of the WF with roughness is linked, according to the literature, to the fact that increased surface roughness generates more surface states that can trap electrons, leading to a decrease in the overall electron density at the surface.

The WF measurements performed by the in vacuo UPS and KPFM were compared using two gold-coated Si substrates. Both methods exhibited WF values that are in close agreement with each other. Additionally, both methods showed that a nanostructured surface exhibits a higher WF in comparison to the wafer, which is explained by the combination of the preferential grain orientation (111) and the compressive stress of the gold coating on the nanostructured Si sample. Finally, the two silicon substrates were exposed to air, and the WF function was measured by air KPFM. The observed WF was found to be 0.4 eV lower than that measured in vacuum, which corresponds with the data presented in the literature. This discrepancy raises concerns about the reliability of air KPFM measurements because of chemical adsorption (carbon contamination) and surface oxidation.

Overall, the paper presents a systematic study of the relationship between the WF and SRs, highlighting two different behaviors depending on the choice of the topography parameters. These results provide new insight into how the morphology of the surface affects the WF of the material, which can find valuable practical applications in electrochemistry, material science, and surface engineering. Further research is needed to develop a deeper theoretical understanding of the WF–SR relationship.

## Supplementary Material



## Data Availability

The raw/processed data that support the findings of this study are available on request from the corresponding author, A.M.D. The data are not publicly available at this time, as they also form part of an ongoing study.

## Notes

SEM image, AFM images, XPS and UPS measurements, statistical metrics, and WF measurements (PDF)
