# Supplementary material for: Topography Influence on Noble Metals’ Work Function Measured In Vacuo by Photoelectron Spectroscopy and Kelvin Probe Force Microscopy
Source: ACS Appl Mater Interfaces. 2026 Jun 9;18(24):34432–41. doi: 10.1021/acsami.6c00932 (PMC13307065; doi:10.1021/acsami.6c00932)
Supplement: Supplementary file 1 [file am6c00932_si_001.pdf]

Topography Influence on Noble Metals' Work Function Measured in Vacuo by Photoelectron Spectroscopy and Kelvin Probe Force Microscopy

Artem M. Dmitriev[a,*], Marcin Kisiel[a], Akash Gupta[a], Laurent Marot[a], Ernst Meyer[a]

[a]*Department of Physics, University of Basel, Klingelbergstrasse 82, CH-4056, Basel, Switzerland*

S1 - SEM image of the sample Si-2 after the deposition of 15 nm Au film.

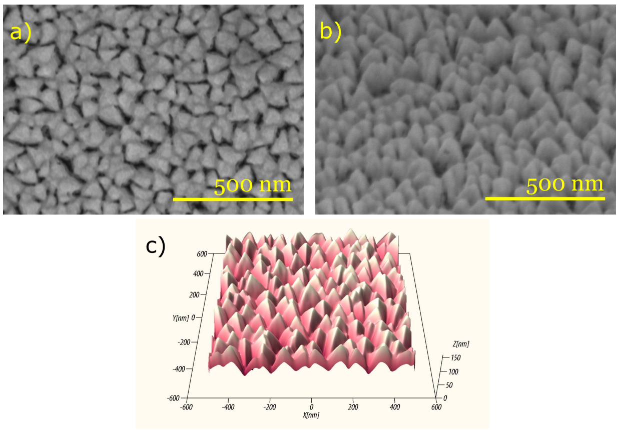

Figure S1: The top (a) and tilted (b) (52°) SEM image of the sample Si-2 after the deposition of 15 nm of Au film. As a substrate, a TipCheck sample (Budget Sensors Ltd.) was used (c) [1]

*Corresponding Author
Email address: artem.dmitriev@unibas.ch (Artem M. Dmitriev)

S2 - The top AFM images of the Si-1 and stainless steel samples.

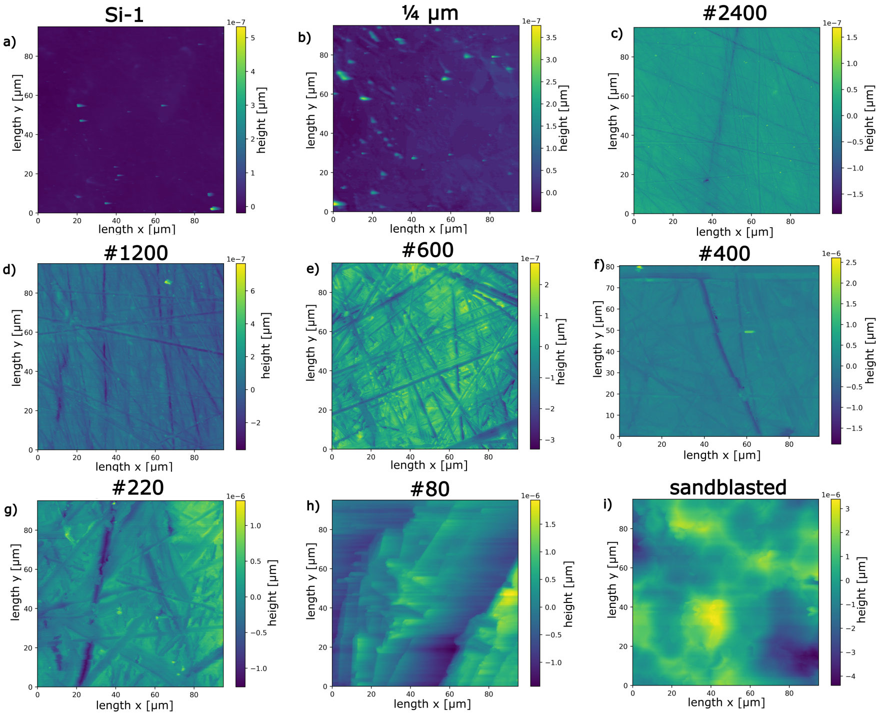

Figure S2: The AFM images of the samples: a) Si-1; stainless steel sample polished with b) 1/4 μm diamond paste; stainless steel samples abraded with c) #2400, d) #1200, e) #600, f) #400, g)#220, h)#80 sandpaper; i) sandblasted sample.

S3 - The XPS and UPS measurements of Au and Ag films

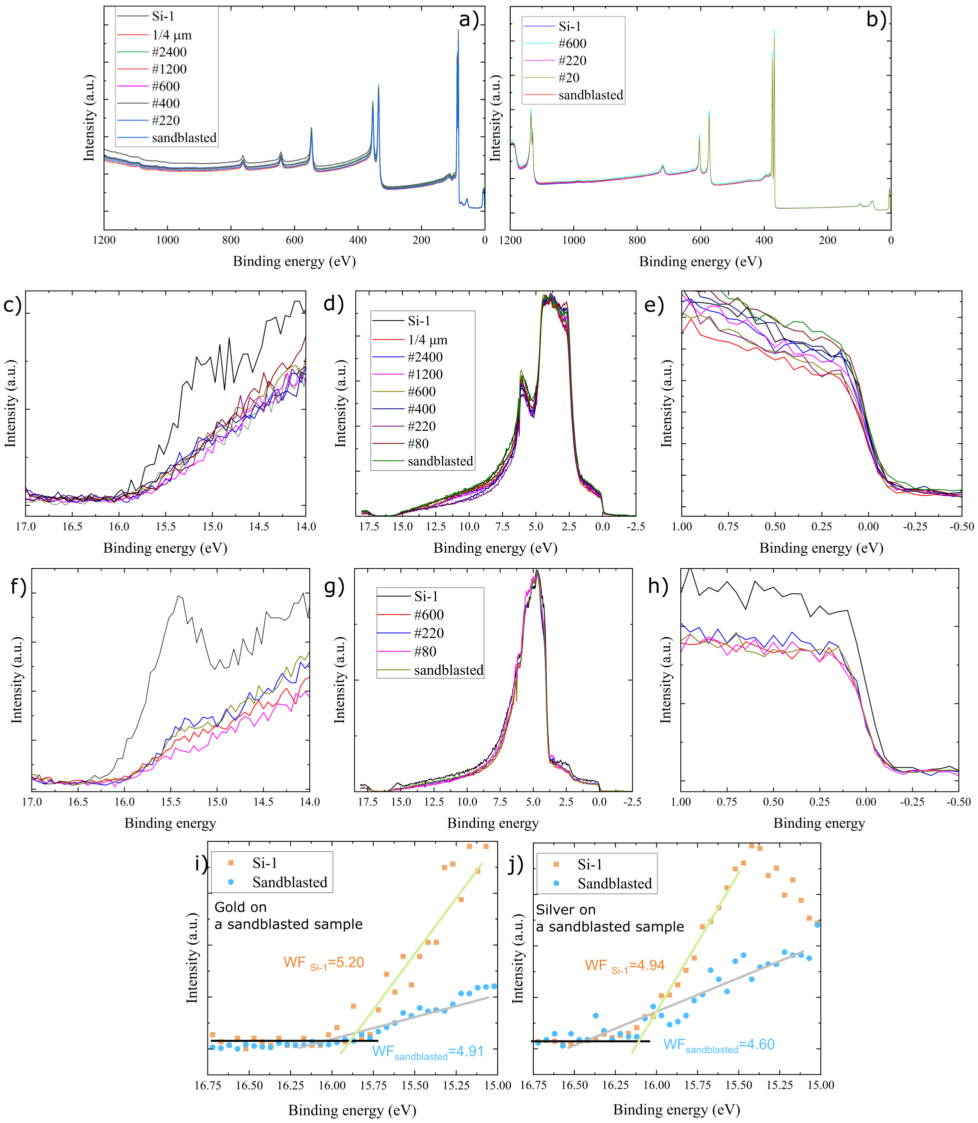

Figure S3: Core level XPS spectra and and UPS measurements of Au and Ag films. a) XPS survey spectra of all the samples with Au deposition; b) XPS survey spectra of all the samples with Ag deposition; c) The cut-off energy region of the UPS spectra of Au samples; d) the UPS spectra of the Au samples; e) the Fermi level region of the UPS spectra of Au samples; f) The cut-off energy region of the UPS spectra of Ag samples; g) the UPS spectra of the Ag samples; h) the Fermi level region of the UPS spectra of Ag samples; i) and j) are examples of the cut-off energy fitting for Au and Ag deposited on the Si-1 and sandblasted samples respectively.

S4 - The WF fitting statistical metrics.

Table S4: Statistical metrics for WF fitting of gold samples.

	Ra	Rq	σ_{rel}		$R_{\Delta a}$	$R_{\Delta q}$	Δm
χ^2	0.00144	0.00138	0.0108	RSS	0.02492	0.02806	0.02104
RSS	0.00866	0.0083	0.02159	Pearson's r	-0.85629	-0.83643	-0.88023
R^2	0.90727	0.91117	0.75067	R^2	0.73324	0.69961	0.7748
Adj. R^2	0.87637	0.88156	0.50135	Adj. R^2	0.69513	0.65669	0.74263

Table S5: Statistical metrics for WF fitting of silver samples.

	Ra	Rq	σ_{rel}		$R_{\Delta a}$	$R_{\Delta q}$	Δm
χ^2	0.00561	0.00558	0.00774	RSS	0.02745	0.02855	0.02454
RSS	0.01683	0.01673	0.02321	Pearson's r	-0.84233	-0.83518	-0.86701
R^2	0.80568	0.80684	0.732	R^2	0.68308	0.67028	0.71666
Adj. R^2	0.7409	0.74246	0.64266	Adj. R^2	0.68308	0.67028	0.71666

S5 - The relationship of R_a and the slope topography parameters ($R_{\Delta a}$, $R_{\Delta q}$, δ_m)

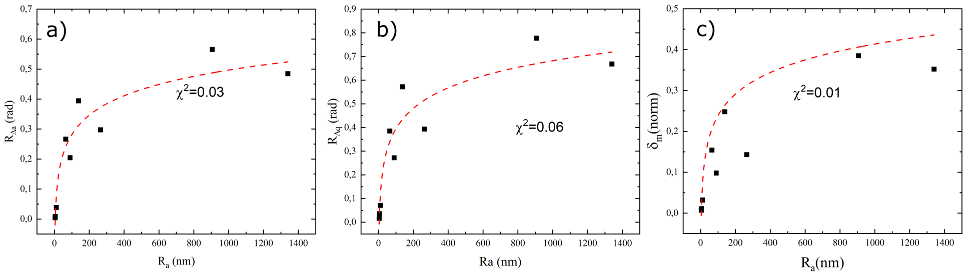

Figure S4: The relationship of average slope roughness a) mean slope roughness ($R_{\Delta a}$), b) RMS slope roughness ($R_{\Delta q}$) and c) mean surface inclination angle (δ_m) as function of the arithmetic roughness R_a. Black squares - experimental data, red dashed line - logarithm fitting $R_i = (a - c)/d + b/d \times ln(R_a)$ (equation (12) in the paper); the parameters of the fitting are presented in tables on the graphs.

Table S6: Parameters of the logarithm law fitting of $R_{\Delta a}$, $R_{\Delta q}$ and δ_m as a function of R_a.

Coefficient	$R_{\Delta a}$	$R_{\Delta q}$	δ_m
(a-c)/d	-0.15	-0.18	-0.11
b/d	0.10	0.13	0.08

S6 - The results of the WF measurements of samples Si-1 and Si-2 performed with air KPFM

Bias voltage (V)

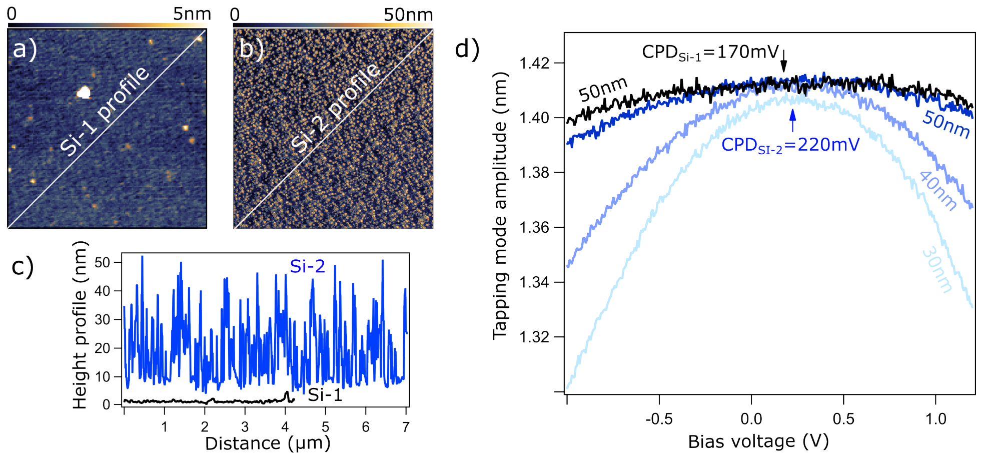

Figure S5: Ambient condition tapping mode AFM images (a,b) and bias spectroscopy parabolas (d) of the Si-1 (a) and Si-2 (b) gold-coated silicon surface performed with SSS-NCL from Nanosensors. The silicon tip work function is equal to WF_{Si} = 4.85 eV. The roughness profiles marked in a) and b) with white lines are shown in c) for the Si-1 (black) and Si-2 (blue) surfaces. On d) the bias spectroscopy parabolas are shown at a few sample distances for the Si-1 (black) and Si-2 (blue surface). The top of the parabola determines the contact potential which is equal to V_{CPD} = 0.17 V and V_{CPD} = 0.22 V for the Si-1 and Si-2 surface, respectively. The corresponding work function under ambient conditions is equal to $WF_{Si\text{-}1}$ = (4.85 + 0.17) eV = 5.02 eV and $WF_{Si\text{-}2}$ = (4.85 + 0.22) eV = 5.07 eV for the Si-1 and Si-2 surface, respectively.

References

[1] BudgetSensors 2025 TipCheck - AFM Calibration Standard accessed: 2025-03-09 URL `https://www.nanoandmore.com/afm-calibration-standard-BUDGETSENSORS-TipCheck`
